# Completeness of variables in Hospital-Based Cancer Registries for prostatic malignant neoplasm

**DOI:** 10.1590/0034-7167-2023-0467

**Published:** 2024-07-29

**Authors:** Wesley Rocha Grippa, Raphael Manhães Pessanha, Larissa Soares Dell’Antonio, Cristiano Soares da Silva Dell’Antonio, Luciane Bresciani Salaroli, Luís Carlos Lopes-Júnior

**Affiliations:** I Universidade Federal do Espírito Santo. Vitoria, Espírito Santo, Brazil; II Secretaria de Estado da Saúde do Espírito Santo, Núcleo Especial de Vigilância Epidemiológica, Instituto Capixaba de Ensino, Pesquisa e Inovação. Vitória, Espírito Santo, Brazil

**Keywords:** Hospital Records, Medical Oncology, Epidemiology, Prostatic Neoplasms, Public Health Surveillance, Registros de Hospitales, Oncología Médica, Epidemiología, Neoplasias de la Próstata, Vigilancia en Salud Pública

## Abstract

**Objectives:**

to analyze the completeness of variables from Hospital-Based Cancer Registries of cases of prostate neoplasm in the Oncology Care Network of a Brazilian state between 2000 and 2020.

**Methods:**

an ecological time series study, based on secondary data on prostate cancer Hospital-Based Cancer Registries prostate. Data incompleteness was classified as excellent (<5%), good (between 5%-10%), fair (10%-20%), poor (20%-50%) and very poor (>50%), according to the percentage of lack of information.

**Results:**

there were 13,519 cases of prostate cancer in the Hospital-Based Cancer Registries analyzed. The variables “family history of cancer” (p<0.001), “alcoholism” (p<0.001), “smoking” (p<0.001), “TNM staging” (p<0.001) had a decreasing trend, while “clinical start of treatment” (p<0.001), “origin” (p=0.008) and “occupation” (p<0.001) indicated an increasing trend.

**Conclusions:**

most Hospital-Based Cancer Registries variables showed excellent completeness, but important variables had high percentages of incompleteness, such as TNM and clinical staging, in addition to alcoholism and smoking.

## RESUMEN

**Objetivos:**

analizar la completitud de las variables del Registro Hospitalario de Cáncer de casos de neoplasias de próstata en la Red de Atención Oncológica de un estado brasileño entre 2000 y 2020.

**Métodos:**

estudio de series de tiempo ecológicas, a partir de datos secundarios sobre el cáncer de próstata Registro Hospitalario de Cáncer próstata. La información incompleta se clasificó como excelente (<5%), buena (entre 5%-10%), regular (10%-20%), mala (20%-50%) y muy mala (>50%), según el porcentaje de falta de información.

**Resultados:**

hubo 13.519 casos de cáncer de próstata en los Registro Hospitalario de Cáncer analizados. Las variables “antecedentes familiares de cáncer” (p<0,001), “alcoholismo” (p<0,001), “tabaquismo” (p<0,001), “estadificación TNM” (p<0,001) tuvieron una tendencia decreciente, mientras que “inicio clínico” de tratamiento” (p<0,001), “origen” (p=0,008) y “ocupación” (p<0,001) indicaron una tendencia creciente.

**Conclusiones:**

la mayoría de las variables Registro Hospitalario de Cáncer mostraron una excelente completitud, pero variables importantes tuvieron altos porcentajes de incompletitud, como el TNM y el estadiaje clínico, además del alcoholismo y el tabaquismo.

## INTRODUCTION

Cancer is a term that covers more than a hundred malignant diseases that have in common uncontrolled cell growth, which can invade adjacent tissues or distant organs^(1)^, claiming the lives of around 9.3 million people annually^(2-3)^. Specifically, prostate cancer is one of the most common cancers in the world, being one of the main causes of premature death in men^(3-4)^.

In Brazil, the Brazilian National Cancer Institute (INCA - *Instituto Nacional do Câncer*) estimates that, for each year of the 2023-2025 triennium, there will be almost 72 thousand new cases of the disease, with an estimated risk of 67.86 new cases and a mortality rate of 13.7 deaths for every 100,000 men^(1)^. In the state of Espírito Santo, prostate cancer is the most common, representing 84.36 new cases for every 100,000 men, according to the latest INCA estimate^(1)^.

Risk factors are well established and include advanced age, ethnicity, genetic factors, family history of cancer and hormonal factors^(1,3,5-6)^, in addition to environmental factors, such as exposure to pesticides, which are still under investigation^(7-9)^. Although there is still little robust evidence for prostate cancer prevention^(5)^, it is possible to reduce the risk by reducing fatty foods, increasing the intake of vegetables and fruits and including physical activity in daily routines^(1,5,10)^.

Hospital-Based Cancer Registries (HBCR) are systematic sources of information, installed in general hospitals or specialized in oncology, with the aim of collecting data regarding diagnosis, treatment and evolution attended in these institutions^(11)^. HBCR provide assistance in collecting and processing information about cancer patients, up to the analysis and dissemination of the bases obtained through consultation of medical records, and, therefore, make a great contribution to Epidemiological Surveillance^(12)^. The information produced makes it possible to analyze the performance and quality of each institution in providing care to cancer patients as well as contributing to prognostic and survival studies^(13)^. They also contribute to individual patient care, as they ensure the follow-up of these patients^(14-15)^.

A recent study by our group on HBCR of a single High Complexity Oncology Care Center (CACON - *Centro de Assistência de Alta Complexidade em Oncologia*) in the state of Espírito Santo showed that most of variables relating to prostate cancer cases, in the time series from 2000 to 2016, had excellent levels of completeness, but several clinical variables, important for a better understanding of the health-disease process, present a high number of missing data, highlighting the need for higher quality data^(16)^. However, an analysis of a more recent time series, that is, until 2020, encompassing the entire Espírito Santo Oncology Care Network, composed of a CACON and seven High Complexity Oncology Care Units (UNACON - *Unidades de Assistência de Alta Complexidade em Oncologia*), in order to direct Cancer Surveillance actions in the Espírito Santo territory regarding HBCR monitoring and assessment of hospitals in the State Oncological Care Network, has not yet been elucidated.

## OBJECTIVES

To analyze the completeness of the HBCR variables of cases of prostate neoplasms in the Oncology Care Network of a Brazilian state between 2000 and 2020.

## METHODS

### Ethical aspects

The study was approved by the *Universidade Federal do Espírito Santo* Health Sciences Center Research Ethics Committee (CEP-CCS-UFES). Patient consent was waived, as this was a retrospective research based on secondary data. Moreover, consent and authorization were obtained from the State Department of Health of Espírito Santo (SESA/ES), based in Vitória, capital, to collect secondary data and access restricted data from this research.

### Study design, period and place

This is an ecological time series study according to STrengthening the Reporting of OBservational studies in Epidemiology (STROBE) recommendations. The study was conducted using secondary data from the HBCR prostate cancer database in the state of Espírito Santo between 2000 and 2020. The secondary data were obtained from SESA/ES Cancer Surveillance and consolidated by INCA.

The Espírito Santo Oncology Care Network covers three health regions: Metropolitan Region; South region; and North/Midwest region^(15)^. This Oncology Care Network is made up of a CACON represented by *Hospital Santa Rita de Cássia*, located in the capital, Vitória, as well as the seven UNACON authorized by the Ministry of Health (MoH): *Hospital Evangélico de Cachoeiro de Itapemirim*, located in the municipality of Cachoeiro from Itapemirim; *Hospital Evangélico de Vila Velha*, located in the city of Vila Velha; *Hospital Universitário Antônio Cassiano de Moraes, Hospital Santa Casa de Misericórdia de Vitória* and *Hospital Estadual Infantil Nossa Senhora da Glória*, located in the capital, Vitória; *Hospital São José*, located in Colatina; and *Hospital Rio Doce*, northern state, located in Linhares. All oncology hospital units in the state have HBCR structured and in operation, with their databases being sent annually to the Brazilian Cancer Hospital Registry Integrating System (SisRHC - *Sistema Integrador do Registro Hospitalar de Câncer Brasileiro*)^(17-18)^. We emphasize that the Hospital Estadual Infantil Nossa Senhora da Glória’s HBCR do not present data regarding diagnoses for prostate cancer.

Data were collected between February and June 2023 from SESA/ES. We chose the period from 2000 to 2020 because it is a more recent period and because all the hospitals that make up the Oncology Care Network in the state of Espírito Santo had already sent, at the time of data collection, records of historical series that we proposed to analyze from the respective HBCR, which were processed and consolidated by the Espírito Santo Epidemiological Surveillance.

### Population, inclusion and exclusion criteria

A total of 13,519 observations (registration of patients diagnosed with prostate cancer) were extracted from the HBCR database in the state of Espírito Santo via SESA/ES in the historical series studied, i.e., from 2000 to 2020, including all cases registered as analytical (whose planning and treatment are carried out in the hospital where registration took place) and non-analytical (those who arrive at the hospital already treated or who do not carry out the recommended treatment, mainly)^(11)^.

### Study protocol

The epidemiological variables contained in the SisRHC tumor registry^(11)^ and analyzed in the present study were: (1) sex; (2) age; (3) place of birth; (4) race/skin color; (5) education; (6) occupation; (7) origin; (8) marital status; (9) history of alcohol consumption; (10) history of tobacco consumption; (11) type of case; (12) date of first hospital consultation; (13) date of first tumor diagnosis; (14) previous diagnosis and treatment; (15) date of start of treatment; (16) screening date; (17) most important basis for tumor diagnosis; (18) primary tumor location; (19) detailed primary tumor location; (20) primary tumor histological type; (21) TNM staging; (22) clinical tumor staging by group (TNM); (23) other staging; (24) pathological TNM staging; (25) main reason for not carrying out antineoplastic treatment in hospital; (26) first treatment received in hospital; (27) disease status at the end of first treatment in hospital; (28) date of death; (29) family history of cancer; (30) source of referral; (31) primary tumor laterality; (32) occurrence of more than one primary tumor; (33) first care clinic; (34) first treatment clinic; (35) examinations relevant to tumor therapy diagnosis and planning; (36) Brazilian National Registry of Health Establishments; (37) Hospital Unit Federative Unit; (38) Hospital Unit municipality.

The HBCR tumor registry form is used to gather information from medical records, provide a case summary and as a data entry document to enter information into the SisRHC computerized databases^(11)^. The content of this form is defined based on the information needs of hospitals with a hospital cancer registry and follows the standardization guidelines recommended by the International Agency for Research on Cancer, validated by consensus by meetings coordinated by INCA^(11)^.

The definition of quality dimensions proposed by Lima *et al*. was used (2009)^(19)^, in which completeness is translated by the proportion of fields filled with non-zero values. Furthermore, as a reference for the analysis of completeness, we adopted the classification proposed by Romero and Cunha (2006)^(20)^. The percentage of missing data was classified as 1 - excellent (<5%), 2 - good (5-10%), 3 - fair (10-20%), 4 - poor (20-50%), or 5 - very poor (≥50%). Thus, the term “completeness” refers to the degree of completion of the analyzed field, measured by the proportion of reports with a field filled in with a different category from those that indicate absence of data. A field filled in the database with the category “ignored”, the numeral zero, unknown date or term indicating absence of data was considered incomplete in this study.

### Analysis of results, and statistics

For statistical analyses, the free software RStudio (version 2023.03.1) and R (version 4.2.2) were used. Completeness description was presented by the relative frequency observed and their respective completeness scores. The Friedman test^(21)^ was used to compare score classifications between years, whereas the Mann-Kendall test^(22-23)^ assessed whether there was a statistically significant temporal trend between the years assessed. A statistical significance level of 0.05 was adopted.

## RESULTS

**Figure 1:**
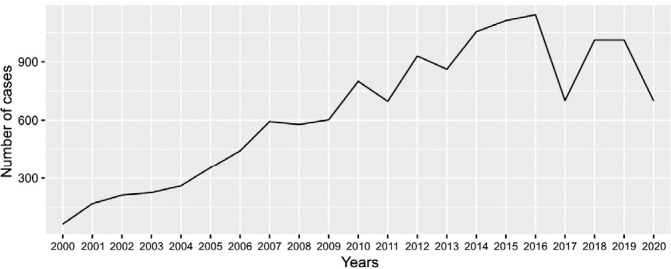
Historical series of the number of prostate cancer cases diagnosed from 2000 to 2020 registered in Hospital-Based Cancer Registries of the state of Espírito Santo (N=13,519)

During the study period, a total of 13,519 cases of prostate cancer recovered from HBCR in the state of Espírito Santo were recorded, as can be seen in Figure 1.

The variable “sex” was the only sociodemographic variable to present 100% completeness, followed by the variable “age”, which presented 0.26% of missing data in 2016, and “origin”, which had incompleteness ranging from 0.20% to 2.34% between 2012 and 2019, therefore, classified as excellent throughout the period studied.

The variable “place of birth” had an average incompleteness of 5.91% in the period, with emphasis on 2000, 2018 and 2020, which presented, respectively, 14.29%, 12.45% and 16.88% of data missing, being classified as fair. The variable “race/skin color” was classified as excellent or good in most of the years studied; however, in 2006 and 2007, it was classified as poor, with 31.67% and 23.78% of incompleteness, respectively. “Marital status” was a variable with an excellent or good score in more than 90% of the years studied, with emphasis on 2012 and 2013, classified as poor, showing incompleteness of 11.08% and 11.48%, respectively.

The variable “education” obtained an excellent score from the years 2000 to 2004, with an average of 2.74% of missing data, however from 2005 to 2020 most years were classified as poor, with emphasis on the year 2010, where almost 50% of observations were missing. Similarly, the variable “occupation” presented an average of 2.20% incompleteness from 2000 to 2004, and from 2005, classified as poor in most of the following years, obtaining in 2018, 22.13% of missing data and classified as poor. Both variables “alcoholism” and “smoking” showed high rates of incompleteness, being classified as very poor and poor in most years of the 2000-2020 historical series studied. Table 1 presents details of year-by-year completeness classifications.

**Table 1:** Percentage of incompleteness and classification of completeness of the Hospital-Based Cancer Registries sociodemographic variables regarding prostate cancer cases in the Oncological Care Network of the state of Espírito Santo from 2000 to 2020 (N = 13,519)

Variable	2000	2001	2002	2003	2004	2005	2006	2007	2008	2009	2010	2011	2012	2013	2014	2015	2016	2017	2018	2019	2020
Sex																					
Incompleteness (%)	0.00	0.00	0.00	0.00	0.00	0.00	0.00	0.00	0.00	0.00	0.00	0.00	0.00	0.00	0.00	0.00	0.00	0.00	0.00	0.00	0.00
Score	1	1	1	1	1	1	1	1	1	1	1	1	1	1	1	1	1	1	1	1	1
Age																					
Incompleteness (%)	0.00	0.00	0.00	0.00	0.00	0.00	0.00	0.00	0.00	0.00	0.00	0.00	0.00	0.00	0.00	0.00	0.26	0.00	0.00	0.00	0.00
Score	1	1	1	1	1	1	1	1	1	1	1	1	1	1	1	1	1	1	1	1	1
Place of birth																					
Incompleteness (%)	14.29	0.00	2.36	6.22	9.23	3.67	3.62	1.01	0.69	3.65	3.88	4.73	5.16	4.76	6.54	3.24	5.08	7.85	12.45	8.79	16.88
Score	3	1	1	2	2	1	1	1	1	1	1	1	2	1	2	1	2	2	3	2	3
Race/skin color																					
Incompleteness (%)	1.59	2.38	0.47	0.00	0.38	10.73	31.67	23.78	9.17	7.81	5.00	12.05	11.29	14.50	6.92	4.32	2.01	1.57	2.57	3.46	3.43
Score	1	1	1	1	1	3	4	4	2	2	2	3	3	3	2	1	1	1	1	1	1
Education																					
Incompleteness (%)	3.17	2.38	2.83	2.22	3.08	29.66	30.77	29.68	32.87	45.02	49.12	26.54	23.55	26.91	26.07	25.09	19.79	12.13	18.87	7.41	12.16
Score	1	1	1	1	1	4	4	4	4	4	4	4	4	4	4	4	3	3	3	2	3
Occupation																					
Incompleteness (%)	0.00	1.19	2.83	3.11	3.85	15.25	15.61	15.68	13.15	7.48	16.12	6.31	14.52	16.24	16.78	18.88	12.52	10.13	22.13	15.71	16.60
Score	1	1	1	1	1	3	3	3	3	2	3	2	3	3	3	3	3	3	4	3	3
Origin																					
Incompleteness (%)	0.00	0.00	0.00	0.44	0.00	0.00	0.00	0.00	0.00	0.00	0.38	0.00	0.22	0.35	0.57	2.34	1.66	0.86	1.38	0.20	0.00
Score	1	1	1	1	1	1	1	1	1	1	1	1	1	1	1	1	1	1	1	1	1
Marital status																					
Incompleteness (%)	0.00	2.38	0.00	0.89	0.00	7.63	3.85	4.05	5.88	6.98	6.12	9.47	11.08	11.48	3.03	3.60	1.31	3.42	1.88	2.08	2.29
Score	1	1	1	1	1	2	1	1	2	2	2	2	3	3	1	1	1	1	1	1	1
Alcoholism																					
Incompleteness (%)	100.00	90.48	96.70	95.11	93.85	82.49	71.95	73.69	71.45	70.10	16.25	9.61	10.32	14.73	27.49	47.84	50.61	41.51	29.55	35.47	33.48
Score	5	5	5	5	5	5	5	5	5	5	3	2	3	3	4	4	5	4	4	4	4
Smoking																					
Incompleteness (%)	98.41	88.10	94.34	94.22	86.15	73.73	61.76	64.42	61.07	58.14	15.62	7.89	10.00	14.27	25.50	45.05	45.97	32.24	22.53	28.16	24.32
Score	5	5	5	5	5	5	5	5	5	5	3	2	3	3	4	4	4	4	4	4	4

*1 - excellent; 2 - good; 3 - fair; 4 - poor; 5 - very poor.*

Concerning clinical variables, the variable “TNM staging” was classified as very poor for all years analyzed, obtaining the worst completeness in 2001, with 97.25% of missing data. Similarly, the variable “family history of cancer” also obtained a very poor score for all years studied, with emphasis on 2000, in which it presented 100% missing data. With a very poor and poor classification for the study period, the variable “clinical tumor staging by group (TNM)” presented incompleteness ranging from 31.45% to 89.62%. Another variable, “pathological TNM staging”, had incompleteness between 14.88% and 39.53%, with scores varying between fair and poor.

The variable “disease status at the end of first treatment in hospital”, from 2000 to 2009, was classified as very poor, with an average of 72.11% of missing observations, but from 2010 onwards it presented better classifications, being poor or fair. and an average of 28.61% incompleteness. The variable “main reason for not carrying out antineoplastic treatment in hospital”, its score varied from excellent to very poor in the period studied, with highlights for the year 2003, which presented incompleteness of just 0.44%, and for the year 2006, reaching almost 72% of missing data. “Referral origin” was a variable classified as poor and fair in most of the years studied, obtaining lower incompleteness rates at the end of the historical series, where in 2020 it presented 7.15% of missing data. The variables “primary tumor laterality” and “examinations relevant to tumor therapy diagnosis and planning” presented an excellent score at the beginning of the study period, being classified as poor and even very poor in the following years.

The variable “previous diagnosis and treatment” and “screening date” presented an excellent classification in almost the entire period, changing the score to good in 2006 and 2007 for the first variable and in 2012 and 2013 for the second. The variable “date of start of treatment” was classified as excellent, except for 2009 to 2012 and 2018, where its score was good or fair.

The other variables in the bank presented excellent scores in all years studied, with emphasis on the variables “type of case”, “date of first consultation”, “primary tumor location”, “detailed primary tumor location”, “primary tumor histological type”, “Brazilian National Registry of Health Establishments”, “Hospital Unit Federative Unit” and “Hospital Unit municipality” which were 100% complete. Table 2 presents in detail and chronologically the incompleteness for clinical variables in the historical series studied.

**Table 2:** Percentage of incompleteness and classification of completeness of the Hospital-Based Cancer Registries clinical variables referring to prostate cancer cases in the Oncological Care Network of the state of Espírito Santo from 2000 to 2020 (N = 13,519)

Variable	2000	2001	2002	2003	2004	2005	2006	2007	2008	2009	2010	2011	2012	2013	2014	2015	2016	2017	2018	2019	2020
Type of case																					
Incompleteness (%)	0.00	0.00	0.00	0.00	0.00	0.00	0.00	0.00	0.00	0.00	0.00	0.00	0.00	0.00	0.00	0.00	0.00	0.00	0.00	0.00	0.00
Score	1	1	1	1	1	1	1	1	1	1	1	1	1	1	1	1	1	1	1	1	1
Date of first consultation																					
Incompleteness (%)	0.00	0.00	0.00	0.00	0.00	0.00	0.00	0.00	0.00	0.00	0.00	0.00	0.00	0.00	0.00	0.00	0.00	0.00	0.00	0.00	0.00
Score	1	1	1	1	1	1	1	1	1	1	1	1	1	1	1	1	1	1	1	1	1
Date of diagnosis																					
Incompleteness (%)	0.00	0.60	0.00	0.00	0.00	0.00	2.49	3.71	2.25	1.99	1.38	2.44	2.04	0.35	0.66	0.09	0.53	0.00	4.15	2.08	0.86
Score	1	1	1	1	1	1	1	1	1	1	1	1	1	1	1	1	1	1	1	1	1
Previous diagnosis and treatment																					
Incompleteness (%)	4.76	0.00	0.00	0.00	0.00	0.00	7.92	5.23	0.35	1.00	2.12	1.00	1.18	0.81	0.38	0.27	0.61	1.00	3.66	1.09	0.14
Score	1	1	1	1	1	1	2	2	1	1	1	1	1	1	1	1	1	1	1	1	1
Date of start of treatment																					
Incompleteness (%)	3.17	0.00	0.47	0.44	0.00	0.00	0.45	0.00	0.00	7.64	14.12	12.20	13.66	4.41	1.99	2.97	3.42	3.42	8.10	2.77	1.00
Score	1	1	1	1	1	1	1	1	1	2	3	3	3	1	1	1	1	1	2	1	1
Screening date																					
Incompleteness (%)	0.00	0.00	0.00	0.00	0.00	0.00	0.00	0.00	0.00	0.00	2.75	0.00	5.48	3.36	2.27	2.34	1.14	2.28	1.88	0.00	0.00
Score	1	1	1	1	1	1	1	1	1	1	1	1	2	2	1	1	1	1	1	1	1
Most important basis for tumor diagnosis																					
Incompleteness (%)	1.59	0.00	0.00	0.44	0.77	0.00	3.85	4.05	2.42	2.33	0.75	1.87	0.86	0.93	1.04	0.27	0.88	1.14	4.15	1.78	0.14
Score	1	1	1	1	1	1	1	1	1	1	1	1	1	1	1	1	1	1	1	1	1
Primary tumor location																					
Incompleteness (%)	0.00	0.00	0.00	0.00	0.00	0.00	0.00	0.00	0.00	0.00	0.00	0.00	0.00	0.00	0.00	0.00	0.00	0.00	0.00	0.00	0.00
Score	1	1	1	1	1	1	1	1	1	1	1	1	1	1	1	1	1	1	1	1	1
Detailed primary tumor location																					
Incompleteness (%)	0.00	0.00	0.00	0.00	0.00	0.00	0.00	0.00	0.00	0.00	0.00	0.00	0.00	0.00	0.00	0.00	0.00	0.00	0.00	0.00	0.00
Score	1	1	1	1	1	1	1	1	1	1	1	1	1	1	1	1	1	1	1	1	1
Primary tumor histological type																					
Incompleteness (%)	0.00	0.00	0.00	0.00	0.00	0.00	0.00	0.00	0.00	0.00	0.00	0.00	0.00	0.00	0.00	0.00	0.00	0.00	0.00	0.00	0.00
Score	1	1	1	1	1	1	1	1	1	1	1	1	1	1	1	1	1	1	1	1	1
TNM staging																					
Incompleteness (%)	95.24	97.26	94.34	88.89	84.62	90.96	50.90	84.65	76.64	81.06	79.25	77.19	79.25	67.17	61.04	56.47	64.10	72.04	70.16	58.89	65.52
Score	5	5	5	5	5	5	5	5	5	5	5	5	5	5	5	5	5	5	5	5	5
Clinical tumor staging by group (TNM)																					
Incompleteness (%)	79.37	85.12	89.62	80.44	74.62	82.77	31.45	67.12	62.80	69.60	60.88	53.08	61.08	51.39	45.69	39.48	37.13	42.94	50.10	40.71	47.50
Score	5	5	5	5	5	5	4	5	5	5	5	5	5	5	4	4	4	4	5	4	4
Other staging																					
Incompleteness (%)	0.00	0.00	0.00	0.00	0.00	0.00	0.00	0.00	0.17	0.00	0.00	0.00	0.11	0.00	0.00	0.09	0.09	0.00	0.00	0.00	0.00
Score	1	1	1	1	1	1	1	1	1	1	1	1	1	1	1	1	1	1	1	1	1
Pathological TNM staging																					
Incompleteness (%)	19.05	21.43	25.47	21.33	30.00	33.05	18.55	16.69	21.97	39.53	26.75	38.59	36.34	31.32	29.10	33.54	34.41	16.41	36.36	15.42	14.88
Score	3	4	4	4	4	4	3	3	4	4	4	4	4	4	4	4	4	3	4	3	3
Main reason for not carrying out antineoplastic treatment in hospital																					
Incompleteness (%)	3.17	4.17	2.83	0.44	1.92	6.50	71.95	7.25	62.28	65.45	71.38	3.01	4.09	6.38	12.51	9.44	11.30	9.56	15.42	4.05	3.72
Score	1	1	1	1	1	2	5	2	5	5	5	1	1	2	3	2	3	2	3	1	1
First treatment received in hospital																					
Incompleteness (%)	1.59	0.00	0.47	0.44	0.00	0.56	0.00	0.00	0.00	0.33	0.50	0.29	0.32	0.46	0.09	2.97	0.26	0.29	6.03	0.30	1.29
Score	1	1	1	1	1	1	1	1	1	1	1	1	1	1	1	1	1	1	2	1	1
Disease status at the end of first treatment in hospital																					
Incompleteness (%)	76.19	85.71	86.79	76.89	74.62	59.89	57.47	59.36	63.32	80.90	36.75	23.82	33.66	19.37	31.00	27.88	17.08	26.68	36.26	26.98	35.19
Score	5	5	5	5	5	5	5	5	5	5	4	4	4	3	4	4	3	4	4	4	4
Date of death																					
Incompleteness (%)	0.00	0.00	0.00	0.00	0.00	0.00	0.23	0.17	0.52	0.00	0.12	0.00	0.22	0.23	0.09	0.00	0.00	0.00	0.10	0.20	0.72
Score	1	1	1	1	1	1	1	1	1	1	1	1	1	1	1	1	1	1	1	1	1
Family history of cancer																					
Incompleteness (%)	100.00	97.02	99.53	96.44	97.69	87.85	83.48	85.50	73.70	71.43	79.88	69.58	74.84	64.50	74.60	72.75	77.58	73.04	71.84	68.38	58.23
Score	5	5	5	5	5	5	5	5	5	5	5	5	5	5	5	5	5	5	5	5	5
Source of referral																					
Incompleteness (%)	30.16	37.50	24.06	13.33	11.92	13.28	26.24	18.55	21.63	17.77	21.88	13.77	19.03	14.62	16.02	7.82	12.08	17.69	8.89	8.50	7.15
Score	4	4	4	3	3	3	4	3	4	3	4	3	3	3	3	2	3	3	2	2	2
Primary tumor laterality																					
Incompleteness (%)	0.00	0.00	0.00	0.00	0.38	0.00	0.45	6.07	12.11	4.65	6.38	10.62	9.46	11.02	12.61	8.99	6.57	2.57	10.08	1.38	4.29
Score	1	1	1	1	1	1	1	2	3	1	2	3	2	3	3	2	2	2	3	3	2
Occurrence of more than one primary tumor																					
Incompleteness (%)	0.00	0.00	0.00	0.00	0.00	0.00	0.00	0.00	0.00	0.00	2.75	0.00	5.48	3.36	2.27	2.25	1.05	2.28	1.88	0.00	0.00
Score	1	1	1	1	1	1	1	1	1	1	1	1	2	1	1	1	1	1	1	1	1
First care clinic																					
Incompleteness (%)	0.00	0.00	0.00	0.00	0.00	0.00	0.00	0.00	0.00	0.00	2.75	0.14	5.48	3.48	2.46	2.79	1.05	2.28	1.88	0.20	0.00
Score	1	1	1	1	1	1	1	1	1	1	1	1	2	1	1	1	1	1	1	1	1
Clinic at the start of treatment																					
Incompleteness (%)	0.00	0.00	0.00	0.00	0.00	0.00	0.00	0.00	0.00	0.00	0.00	0.00	0.32	0.46	0.19	0.36	0.35	0.43	5.93	0.20	0.86
Score	1	1	1	1	1	1	1	1	1	1	1	1	1	1	1	1	1	1	2	1	1
Examinations relevant to tumor therapy diagnosis and planning																					
Incompleteness (%)	0.00	0.60	0.00	0.00	4.23	1.13	19.00	18.04	24.39	25.42	12.50	6.17	8.06	5.10	9.76	10.70	4.12	3.85	9.49	7.91	10.59
Score	1	1	1	1	1	1	3	3	4	4	3	2	2	2	2	3	1	1	2	2	3
Brazilian National Registry of Health Establishments																					
Incompleteness (%)	0.00	0.00	0.00	0.00	0.00	0.00	0.00	0.00	0.00	0.00	0.00	0.00	0.00	0.00	0.00	0.00	0.00	0.00	0.00	0.00	0.00
Score	1	1	1	1	1	1	1	1	1	1	1	1	1	1	1	1	1	1	1	1	1
Hospital Unit Federative Unit																					
Incompleteness (%)	0.00	0.00	0.00	0.00	0.00	0.00	0.00	0.00	0.00	0.00	0.00	0.00	0.00	0.00	0.00	0.00	0.00	0.00	0.00	0.00	0.00
Score	1	1	1	1	1	1	1	1	1	1	1	1	1	1	1	1	1	1	1	1	1
Hospital Unit municipality																					
Incompleteness (%)	0.00	0.00	0.00	0.00	0.00	0.00	0.00	0.00	0.00	0.00	0.00	0.00	0.00	0.00	0.00	0.00	0.00	0.00	0.00	0.00	0.00
Score	1	1	1	1	1	1	1	1	1	1	1	1	1	1	1	1	1	1	1	1	1

*1 - excellent; 2 - good; 3 - fair; 4 - poor; 5 - very poor.*

Regarding the comparison of the scores of the HBCR epidemiological variables in the state of Espírito Santo, the Friedman test showed that there was no significant difference (*p* value = 0.324) in score classification; therefore, classification was similar between 2000 and 2020.

In Table 3, the Mann-Kendall test shows significant trends towards a decrease in incompleteness for the variables “family history of cancer”, “alcoholism”, “smoking”, “source of referral”, “TNM staging”, “clinical tumor staging by group (TNM)” and “disease status at the end of first treatment in hospital”. The variables “place of birth”, “first care clinic”, “clinic at the start of treatment”, “origin”, “primary tumor laterality” and “occupation” showed an increasing trend in the incompleteness rate. The variables that presented 100% completeness in all years studied were not included in the Mann-Kendall test and, therefore, do not appear in Table 3.

**Table 3:** Analysis of the trend of incompleteness of the Hospital-Based Cancer Registries epidemiological and clinical variables regarding prostate cancer cases in the Oncological Care Network of the state of Espírito Santo from 2000 to 2020 (N = 13.519)

Variable	S	*p* value^*^	Trend
Age	12	0,364	Not significant
Place of birth	80	0,017	Increase
Race/skin color	-2	0,976	Not significant
Education	0	1,000	Not significant
First care clinic	73	0,018	Increase
Clinic at the start of treatment	118	< 0,001	Increase
Family history of cancer	-148	< 0,001	Decrease
Alcoholism	-122	< 0,001	Decrease
Smoking	-128	< 0,001	Decrease
Origin	81	0,008	Increase
Source of referral	-106	0,002	Decrease
Examinations relevant to tumor therapy diagnosis and planning	47	0,164	Not significant
Marital status	29	0,397	Not significant
Previous diagnosis and treatment	27	0,428	Not significant
Most important basis for tumor diagnosis	29	0,397	Not significant
Primary tumor laterality	88	0,008	Increase
Occurrence of more than one primary tumor	54	0,065	Not significant
TNM staging	-133	< 0,001	Decrease
Clinical tumor staging by group (TNM)	-134	< 0,001	Decrease
Other staging	17	0,477	Not significant
Pathological TNM staging	14	0,695	Not significant
Main reason for not carrying out antineoplastic treatment in hospital	38	0,264	Not significant
First treatment received in hospital	33	0,330	Not significant
Disease status at the end of first treatment in hospital	-126	< 0,001	Decrease
Occupation	122	< 0,001	Increase
Date of diagnosis	37	0,271	Not significant
Screening date	54	0,065	Not significant
Date of start of treatment	53	0,113	Not significant
Date of death	52	0,095	Not significant

* *For significance, p value < 0.05.*

**Figure 2:**
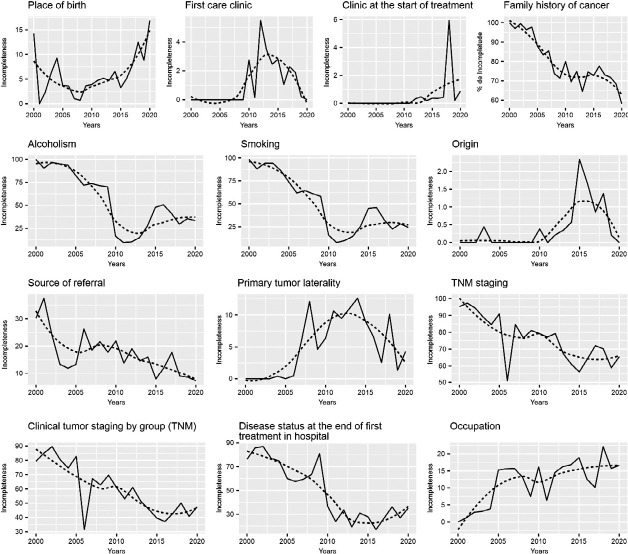
Trend of incompleteness of sociodemographic and clinical variables with a significant trend according to the Hospital-Based Cancer Registries Mann-Kendall test regarding prostate cancer cases in the Oncological Care Network of the state of Espírito Santo from 2000 to 2020 (N = 13,519)

Figure 2 shows the graphs of historical series from 2000 to 2020 with the percentage of incompleteness of the variables that showed significant trends according to the Mann-Kendall test for the period studied. Time series with incomplete data are represented by solid lines, while dashed lines represent the temporal trend.

## DISCUSSION

The results showed that, with regard to cases of malignant prostatic neoplasm in the state of Espírito Santo recovered and analyzed in HBCR, the majority of epidemiological variables were classified as having excellent and/or good completeness, highlighting the variables “sex”, “age”, “origin”, “date of first consultation”, “date of diagnosis”, “previous diagnosis and treatment”, “most important basis for tumor diagnosis”, “primary tumor location”, “detailed primary tumor location”, “primary tumor histological type” and “first treatment received in hospital”. However, other variables were classified in some years as fair and poor, such as “place of birth”, “race/skin color”, “education”, “occupation”, “marital status”, “date of start of treatment”, “examinations relevant to tumor therapy diagnosis and planning”. Furthermore, there was weakness in the information on important clinical-epidemiological variables, with incompleteness above 50%, such as “TNM staging”, “clinical tumor staging by group (TNM)”, “family history”, in addition to “alcoholism” and “smoking”. Supporting our results, a study carried out with data from HBCR in the state of Mato Grosso showed that the variables “education”, “TNM staging”, “family history of cancer”, “alcoholism” and “smoking” exhibited incompleteness above 50%^(24)^.

The variables “sex” and “age” presented completeness classified as excellent in the analyzed database, as found in other studies from HBCR in other Brazilian states^(13,15-16,24)^. It is believed that the low interpretative subjectivity required to record this information corroborates the reason for this good result.

The variable “place of birth” obtained 5.91% of incompleteness, leaving it with a good score, but it showed a tendency to increase in incompleteness in the period analyzed. In other studies conducted in HBCR in the state of Espírito Santo, this variable presented an average incompleteness of 10.33%^(15)^ and 3.51%^(16)^.

“Race/skin color” is an important variable in the study of prostate cancer, as some ethnicities are risk factors for the development of this cancer, such as Africans and Asians, presenting higher incidence rates and shorter survival times for this neoplasm^(5,25)^. In other words, this variable merely transcends a biological distinction. In fact, it encompasses a complexity that represents a set of economic and cultural connotations, which denote inequalities in access to medical care, especially in the context of cancer diagnosis and treatment. Our findings support other research carried out in Brazil^(15,24,26-27)^. It is important to highlight that the lack of completeness in the collection of this variable, combined with possible erroneous records, makes it difficult to obtain a clear understanding of the real need for health promotion and disease prevention programs in vulnerable communities^(27)^. Additionally, the variable that considers race/ethnicity gains relevance by expanding debates to health inequities and individual, social and political-programmatic vulnerability^(15,28-29)^.

“Education” was classified as poor in more than 50% of the study period, with an average incompleteness of 31%, a result similar to that found in other studies^(12,15,24,27,30)^. The result found in HBCR of *Hospital Santa Rita de Cássia*, the only CACON in the state of Espírito Santo, presented 9.12% of missing data, which implies that the other HBCR in Espírito Santo have greater incompleteness for this variable^(16)^. This variable has a great impact on patients’ prognosis, and its low completeness is of clinical and epidemiological relevance^(15)^.

The variable “occupation” presented, at the beginning of historical series, an excellent classification, however, from 2005 to 2020, there was an increase in the percentage of missing data, becoming a fair classification, with an average incompleteness of 14.57% in the period. In a study carried out in HBCR in 21 Brazilian states regarding the occupation variable, 46% of missing observations were identified^(31)^. Other studies find similar percentages^(12,15-16,24,27)^.

The variables “TNM staging”, “clinical tumor staging by group (TNM)” and “pathological TNM staging” presented a poor or very poor completeness score in almost all years. These results corroborate other studies using data from HBCR across Brazil^(12,14-16,32)^. On the other hand, a study using a database from a public hospital in São Paulo showed the variable “TNM staging” with excellent levels of completeness^(27)^. Staging variables are extremely important, as they provide information on the extent of the disease. This information helps in defining the therapeutic plan for people with cancer, which facilitates the standardization of procedures and the exchange of experiences between institutions that offer cancer treatment^(11,15,24,33)^.

The variables “alcoholism” and “smoking” were classified as poor or very poor in almost the entire study period. This is a poor result, given the carcinogenic potential of alcohol and tobacco^(34)^. Furthermore, the variable “family history of cancer” was also classified as very poor in all years of the period, representing almost 80% of average incompleteness. This probably occurred because they are optional variables in the tumor form, and their completion varies substantially between hospital institutions. Such incompleteness is a worrying factor, as this variable is a risk factor for prostate cancer^(2,5-6,35-37)^.

### Study limitations

The present study has some limitations, such as the exclusive use of data obtained from all HBCR in a single Brazilian state. Consequently, caution must be taken when interpreting the findings in relation to their external validity and generalization to other Brazilian states and regions. Although HBCR provide valuable information about the quality of services offered, they do not comprehensively represent the underlying regional or national cancer epidemiology.

### Contributions to nursing, health or public policy

To the best of our knowledge, this is the first study in a recent historical series that reports completeness of HBCR epidemiological variables on cases of malignant prostatic neoplasm across the Espírito Santo (ES) Oncological Care Network between 2000 and 2020, bringing valuable information for Epidemiological Surveillance and, specifically, for Cancer Surveillance in the Espírito Santo territory. It should be noted that, in 80% of countries, there is a growing trend in premature mortality from cancer, which is impacting the achievement of target 3.4 of the Sustainable Development Goals, which refers to the reduction of at least one third in premature mortality due to chronic non-communicable diseases by 2030^(38)^. Thus, the importance of implementing, maintaining, updating and making available HBCR data is evident for a better understanding of cancer overview for its monitoring and control.

## CONCLUSIONS

Summing up, we verified that, in fact, most of the revised HBCR epidemiological variables in the state of Espírito Santo, Brazil, were classified with excellent completeness, although important variables, such as “TNM staging” and “clinical tumor staging by group (TNM)”, had high incompleteness rates for all years between 2000 and 2020. There is a pressing need for consistent and high-quality HBCR data to better monitoring of epidemiological variables in the tumor registry. HBCR contributions can greatly contribute to the structuring, formulation and planning of public policies aimed at improving early diagnosis, treatment and quality of life of the population.

## References

[1] Instituto Nacional de Câncer José Alencar Gomes da Silva (2022). Estimativa 2023: incidência do câncer no Brasil.

[2] World Health Organization (WHO) (2022). Health Statistics and Information Systems: Disease Burden and Mortality Estimates.

[3] Sung H, Ferlay J, Siegel RL, Laversanne M, Soerjomataram I, Jemal A (2021). Global Cancer Statistics 2020: GLOBOCAN Estimates of Incidence and Mortality Worldwide for 36 Cancers in 185 Countries. CA Cancer J Clin.

[4] Ferlay J. (2020). Global Cancer Observatory: cancer today.

[5] Rawla P. (2019). Epidemiology of Prostate Cancer. World J Oncol.

[6] Siegel RL, Miller KD, Wagle NS, Jemal A. (2023). Cancer statistics: 2023. CA Cancer J Clin.

[7] Koutros S, Beane Freeman LE, Berndt SI, Andreotti G, Lubin JH, Sandler DP (2010). Pesticide use modifies the association between genetic variants on chromosome 8q24 and prostate cancer. Cancer Res.

[8] Deziel NC, Beane Freeman LE, Hoppin JA, Thomas K, Lerro CC, Jones RR (2019). An algorithm for quantitatively estimating non-occupational pesticide exposure intensity for spouses in the Agricultural Health Study. J Expo Sci Environ Epidemiol.

[9] Dutra LS, Ferreira AP, Horta MAP, Palhares PR. (2020). Use of pesticides and cancer mortality in monoculture regions. Saúde Debate.

[10] American Cancer Society (2021). Prostate Cancer Stages.

[11] Instituto Nacional de Câncer (INCA) (2010). Registros Hospitalares de Câncer: planejamento e gestão.

[12] Pinto IV, Ramos DN, Costa MCE, Ferreira CB, Rebelo MS. (2012). Completeness and consistency of data in hospital-based cancer registries in Brazil. Cad Saude Colet.

[13] Edwards D, Bell J. (2000). Cancer registries-future development and uses in Britain. J Public Health Med.

[14] Parkin DM. (2008). The role of cancer registries in cancer control. Int J Clin Oncol.

[15] Lopes-Júnior LC, Dell’Antonio LS, Pessanha RM, Dell’Antonio C, Silva MI, Souza TM (2022). Completeness and Consistency of Epidemiological Variables from Hospital-Based Cancer Registries in a Brazilian State. Int J Environ Res Public Health.

[16] Grippa WR, Dell'Antonio LS, Salaroli LB, Lopes-Júnior LC. (2023). Incompleteness trends of epidemiological variables in a Brazilian high complexity cancer registry: an ecological time series study. Medicine.

[17] Pereira LD, Schuab SIPC, Pessanha RM, Amorim MAC, Zandonade E, Lopes-Júnior LC., Silva FJG, Sales JCS, Galiza FT (2020). Neoplasias malignas e a importância dos registros de câncer. Políticas, Epidemiologia e Experiências no Sistema Único de Saúde (SUS): possibilidades e desafios do Cenário Brasileiro.

[18] Secretaria do Estado do Espírito Santo (SESA) (2017). Informativo Vigilância do Câncer: n.17.

[19] Lima CR, Schramm JM, Coeli CM, Silva ME. (2009). [Review of data quality dimensions and applied methods in the evaluation of health information systems]. Cad Saude Publica.

[20] Romero DE, Cunha CB. (2006). [Quality of socioeconomic and demographic data in relation to infant mortality in the Brazilian Mortality Information System (1996/2001)]. Cad Saude Publica.

[21] Hollander M, Wolfe DA. (1973). Nonparametric Statistical Methods.

[22] Kendall MG. (1975). Rank correlation methods.

[23] Mann HB. (1945). Nonparametric Tests Against Trend. Econometrica.

[24] Oliveira JCS, Azevedo EFS, Caló RS, Atanaka M, Galvão ND, Silva AMC. (2021). Registros Hospitalares de Câncer de Mato Grosso: análise da completitude e da consistência. Cad Saude Colet.

[25] Wu D, Yang Y, Jiang M, Yao R. (2022). Competing risk of the specific mortality among Asian-American patients with prostate cancer: a surveillance, epidemiology, and end results analysis. BMC Urol.

[26] Felix JD, Zandonade E, Amorim MH, Castro DS. (2012). Evaluation of the plenitude of epidemiological variables of the Information System on Mortality of women with deaths from breast cancer in the Southeast Region: Brazil (1998 - 2007). Cien Saude Colet.

[27] Brandão-Souza C, Amorim MHC, Zandonade E, Fustinoni SM, Schirmer J. (2019). Completeness of medical records of elderly women with breast cancer: a trend study. Acta Paul Enferm.

[28] Ayres de CM, Franca I, Calazans GJ, Saletti HC. (2003). In: Promoção da saúde: conceitos, reflexões, tendências.

[29] Lopes-Júnior LC, Lima RAG. (2019). Cancer care and interdisciplinary practice. Cad Saude Publica.

[30] Dell'Antonio LS, Leite FMC, Dell'Antonio CSDS, Souza CB, Garbin JRT, Santos APBD (2023). Completeness and quality of information about death from COVID-19 in a Brazilian state: a descriptive population-based register study. Medicine.

[31] Grabois MF, Souza MC, Guimarães RM, Otero UB. (2014). Completeness of information occupation in hospital cancer records in Brazil: basis for surveillance of work-related cancer. Rev Bras Cancerol.

[32] Rebelo PAP, Lima RGM, Souto RM. (2000). Registros Hospitalares de Cancer: rotinas e procedimentos.

[33] O'Sullivan B, Brierley J, Byrd D, Bosman F, Kehoe S, Kossary C (2017). The TNM classification of malignant tumours-towards common understanding and reasonable expectations. Lancet Oncol.

[34] Leite RB, Marinho ACO, Costa BL, Laranjeira MBV, Araújo KDT, Cavalcanti AFM. (2021). The influence of tobacco and alcohol in oral cancer: literature review. J Bras Patol Med Lab.

[35] Flória-Santos M, Lopes-Júnior LC, Alvarenga LM, Ribeiro MS, Ferraz VE, Nascimento LC (2016). Self-reported cancer family history is a useful tool for identification of individuals at risk of hereditary cancer predisposition syndrome at primary care centers in middle-income settings: a longitudinal study. Genet Mol Biol.

[36] Silva TB, Macdonald DJ, Ferraz VE, Nascimento LC, Santos CB, Lopes-Júnior LC (2013). Perception of cancer causes and risk, family history and preventive behaviors of users in oncogenetic counseling. Rev Esc Enferm USP.

[37] Pessanha RM, Schuab SIPC, Nunes KZ, Lopes-Júnior LC. (2022). Use of family history taking for hereditary neoplastic syndromes screening in primary health care: a systematic review protocol. PLoS One.

[38] World Health Organization (WHO) (2020). WHO report on cancer: setting priorities, investing wisely and providing care for all.

