# Supplementary material for: Completeness of variables in Hospital-Based Cancer Registries for prostatic malignant neoplasm
Source: Rev Bras Enferm. 2024 Jul 29;77(3):e20230467. doi: 10.1590/0034-7167-2023-0467 (PMC11290746; doi:10.1590/0034-7167-2023-0467)
Supplement: Translation

# Translation: Completude das variáveis dos Registros Hospitalares de Câncer de neoplasia maligna prostática

**DOI:** 10.1590/0034-7167-2023-0467pt

**Authors:** Wesley Rocha Grippa, Raphael Manhães Pessanha, Larissa Soares Dell’Antonio, Cristiano Soares da Silva Dell’Antonio, Luciane Bresciani Salaroli, Luís Carlos Lopes-Júnior

**Affiliations:** I Universidade Federal do Espírito Santo. Vitoria, Espírito Santo, Brasil; II Secretaria de Estado da Saúde do Espírito Santo, Núcleo Especial de Vigilância Epidemiológica, Instituto Capixaba de Ensino, Pesquisa e Inovação. Vitória, Espírito Santo, Brasil

**Keywords:** Registros Hospitalares, Oncologia, Epidemiologia, Neoplasias da Próstata, Vigilância em Saúde Pública

## Abstract

**Objetivos:**

analisar a completude das variáveis dos Registros Hospitalares de Câncer dos casos de neoplasia prostática da Rede de Atenção Oncológica de um estado brasileiro entre 2000 e 2020.

**Métodos:**

estudo ecológico de séries temporais, baseados em dados secundários de câncer de próstata dos Registros Hospitalares de Câncer. A incompletude dos dados foi classificada como excelente (<5%), boa (entre 5%-10%), regular (10%-20%), ruim (20%-50%) e muito ruim (>50%), de acordo com o percentual de ausência de informação.

**Resultados:**

foram analisados 13.519 casos de câncer próstata. As variáveis “histórico familiar de câncer” (p<0,001), “alcoolismo” (p<0,001), “tabagismo” (p<0,001), “estadiamento TNM” (p<0,001) tiveram tendência de decréscimo, enquanto que “clínica do início do tratamento” (p<0,001), “procedência” (p=0,008) e “ocupação” (p<0,001) indicaram tendência crescente.

**Conclusões:**

a maioria das variáveis dos Registros Hospitalares de Câncer apresentou completude excelente, porém importantes variáveis tiveram altos percentuais de incompletude, como estadiamento TNM e clínico, além de alcoolismo e tabagismo.

## INTRODUÇÃO

Câncer é um termo que abrange mais de uma centena de doenças malignas que têm em comum o crescimento celular descontrolado, que podem invadir tecidos adjacentes ou órgãos a distância^(1)^, ceifando a vida de cerca de 9,3 milhões de pessoas anualmente^(2-3)^. Especificamente, o câncer de próstata é um dos mais incidentes no mundo, sendo uma das principais causas de morte prematura em homens^(3-4)^.

No Brasil, o Instituto Nacional do Câncer (INCA) estima que, para cada ano do triênio 2023-2025, ocorrerão quase 72 mil novos casos da doença, com risco estimado de 67,86 novos casos e taxa de mortalidade de 13,7 óbitos para cada 100 mil homens^(1)^. No estado do Espírito Santo, o câncer de próstata é o mais incidente, representando 84,36 novos casos para cada 100 mil homens, segundo a última estimativa do INCA^(1)^.

Os fatores de risco estão bem estabelecidos, e incluem idade avançada, etnia, fatores genéticos, história familiar de câncer e fatores hormonais^(1,3,5-6)^, além de fatores ambientais, como exposição a agrotóxicos que ainda estão sob investigação^(7-9)^. Embora ainda existam poucas evidências robustas para prevenção do câncer de próstata^(5)^, é possível diminuir o risco com a redução de alimentos gordurosos, aumento da ingesta de vegetais e frutas e inclusão de atividade física nas rotinas diárias^(1,5,10)^.

Os Registros Hospitalares de Câncer (RHC) são fontes sistemáticas de informações instalados em hospitais gerais ou especializados em oncologia, com o intuito de coletar dados referentes ao diagnóstico, tratamento e evolução atendidos nessas instituições^(11)^. Os RHC prestam assistência na coleta e no processamento de informações sobre os pacientes com câncer, até a análise e divulgação das bases obtidas por meio de consulta aos prontuários, e, portanto, apresentam grande contribuição para a Vigilância Epidemiológica^(12)^. A informação produzida permite analisar o desempenho e a qualidade de cada instituição na assistência ao paciente oncológico, bem como contribuir com estudos prognósticos e de sobrevida^(13)^. Contribuem, ainda, na atenção ao paciente individualmente, uma vez que asseguram o seguimento desses pacientes^(14-15)^.

Estudo recente do nosso grupo sobre os RHC de um único Centro de Assistência de Alta Complexidade em Oncologia (CACON) no estado do Espírito Santo mostrou que grande parte das variáveis referentes aos casos de câncer de próstata, na série temporal de 2000 a 2016, teve excelentes níveis de completude, porém diversas variáveis clínicas, importantes para uma melhor compreensão do processo de saúde-doença, apresentam elevado número de dados faltantes, ressaltando a necessidade de ter dados com mais qualidade^(16)^. Contudo, uma análise de uma série temporal mais recente, ou seja, até 2020, englobando toda a Rede de Atenção Oncológica do estado do Espírito Santo, composta por um CACON e sete Unidades de Assistência de Alta Complexidade em Oncologia (UNACON), de modo a direcionar as ações de Vigilância do Câncer no território capixaba quanto ao monitoramento e à avaliação dos RHC dos hospitais da Rede Estadual de Atenção Oncológica, ainda não foi elucidada.

## OBJETIVOS

Analisar a completude das variáveis dos RHC dos casos de neoplasia prostática da Rede de Atenção Oncológica de um estado brasileiro entre 2000 e 2020.

## MÉTODOS

### Aspectos éticos

O estudo foi aprovado pelo Comitê de Ética em Pesquisa do Centro de Ciências da Saúde da Universidade Federal do Espírito Santo (CEP-CCS-UFES). O consentimento do paciente foi dispensado, por se tratar de pesquisa retrospectiva com base em dados secundários. Além disso, obtiveram-se a anuência e a autorização da Secretaria de Estado da Saúde do Espírito Santo (SESA/ES), com sede em Vitória, capital, para a coleta de dados secundários e acesso aos dados restritos desta pesquisa.

### Desenho, período e local do estudo

Trata-se de estudo ecológico de séries temporais segundo as recomendações do *STrengthening the Reporting of OBservational studies in Epidemiology* (STROBE). O estudo foi conduzido a partir de dados secundários provenientes do banco de dados de câncer de próstata dos RHC do estado do Espírito Santo entre os anos de 2000 e 2020. Os dados secundários foram obtidos na Vigilância do Câncer da SESA/ES e consolidados pelo INCA.

A Rede de Atenção à Oncologia do Espírito Santo abrange três regiões de saúde: Região Metropolitana; Região Sul; e Região Norte/Centro^(15)^. Essa Rede de Atenção Oncológica é composta por um CACON representado pelo Hospital Santa Rita de Cássia, localizado na capital, Vitória, bem como pelas sete UNACON habilitadas pelo Ministério da Saúde (MS): Hospital Evangélico de Cachoeiro de Itapemirim, localizado no município de Cachoeiro de Itapemirim; Hospital Evangélico de Vila Velha, localizado na cidade de Vila Velha; Hospital Universitário Antônio Cassiano de Moraes, Hospital Santa Casa de Misericórdia de Vitória e Hospital Estadual Infantil Nossa Senhora da Glória, localizados na capital, Vitória; Hospital São José, localizado em Colatina; e Hospital Rio Doce, no norte do estado, localizado em Linhares. Todas as unidades hospitalares oncológicas do estado apresentam os RHC estruturados e em funcionamento, com envio de suas bases anualmente ao Sistema Integrador do Registro Hospitalar de Câncer Brasileiro (SisRHC)^(17-18)^. Ressaltamos que os RHC do Hospital Estadual Infantil Nossa Senhora da Glória não apresentam dados referentes a diagnósticos para câncer de próstata.

Os dados foram coletados entre fevereiro e junho de 2023 junto à SESA/ES. Escolhemos o período de 2000 a 2020 por se tratar de um período mais recente e pelo fato de que todos os hospitais que compõem a Rede de Atenção Oncológica do estado do Espírito Santo já haviam enviado, até o momento da coleta de dados, os registros da séria histórica que propusemos analisar dos respectivos RHC, os quais foram processados pela Vigilância Epidemiológica do estado do Espírito Santo e consolidados.

### População, critérios de inclusão e exclusão

Foram extraídas do banco de dados dos RHC do estado do Espírito Santo via SESA/ES 13.519 observações (registro de pacientes diagnosticados com câncer de próstata) na série histórica estudada, ou seja, de 2000 a 2020, sendo incluídos todos os casos registrados como analíticos (cujo planejamento e tratamento são realizados no hospital onde se deu o registro) e não analíticos (aqueles que chegam ao hospital já tratados ou que não realizam o tratamento preconizado, principalmente)^(11)^.

### Protocolo do estudo

As variáveis epidemiológicas contidas na ficha do tumor SisRHC^(11)^ e analisadas no presente estudo foram: (1) sexo; (2) idade; (3) local de nascimento; (4) raça/cor da pele; (5) escolaridade; (6) ocupação; (7) procedência; (8) estado conjugal; (9) histórico de consumo de álcool; (10) histórico de consumo de tabaco; (11) tipo de caso; (12) data da primeira consulta no hospital; (13) data do primeiro diagnóstico do tumor; (14) diagnóstico e tratamento anterior; (15) data do início do tratamento; (16) data da triagem; (17) base mais importante para o diagnóstico do tumor; (18) localização primária do tumor; (19) localização primária detalhada do tumor; (20) tipo histológico do tumor primário; (21) estadiamento TNM; (22) estadiamento clínico do tumor por grupo (TNM); (23) outro estadiamento; (24) estadiamento TNM patológico; (25) principal razão para a não realização do tratamento antineoplásico no hospital; (26) primeiro tratamento recebido no hospital; (27) estado da doença ao final do primeiro tratamento no hospital; (28) data do óbito; (29) histórico familiar de câncer; (30) origem do encaminhamento; (31) lateralidade do tumor primário; (32) ocorrência de mais de um tumor primário; (33) clínica do primeiro atendimento; (34) clínica do primeiro tratamento; (35) exames relevantes para o diagnóstico e planejamento da terapêutica do tumor; (36) Cadastro Nacional de Estabelecimentos de Saúde; (37) Unidade Federativa da Unidade Hospitalar; (38) município da Unidade Hospitalar.

A ficha de registro de tumor dos RHC é utilizada para reunir informações do prontuário, fornecer um resumo do caso e como um documento de entrada de dados para inserir informações nos bancos de dados informatizados do SisRHC^(11)^. O conteúdo desse formulário é definido com base nas necessidades de informação dos hospitais com registro hospitalar de câncer, e segue as diretrizes de padronização recomendadas pela Agência Internacional de Pesquisa em Câncer da Organização Mundial da Saúde (*International Agency for Research on Cancer*), validadas em consenso por reuniões coordenadas pelo INCA^(11)^.

Foi utilizada a definição de dimensões da qualidade proposta por Lima *et al*. (2009)^(19)^, na qual a completude é traduzida pela proporção de campos preenchidos com valores não nulos. Além disso, como referência para a análise da completude, adotamos a classificação proposta por Romero e Cunha (2006)^(20)^. A porcentagem de dados ausentes foi classificada como 1 - excelente (<5%), 2 - boa (5-10%), 3 - regular (10-20%), 4 - ruim (20-50%) ou 5 - muito ruim (≥50%). Assim, o termo “completude” refere-se ao grau de preenchimento do campo analisado, medido pela proporção de notificações com campo preenchido com categoria diferente daquelas que indicam ausência de dados. Um campo preenchido no banco de dados com a categoria “ignorado”, o numeral zero, data desconhecida ou termo indicando ausência de dados foi considerado incompleto neste estudo.

### Análise dos resultados e estatística

Para análises estatísticas, utilizaram-se os *softwares* livres RStudio (versão 2023.03.1) e R (versão 4.2.2). A descrição da completude foi apresentada pela frequência relativa observada e seus respectivos escores de completude. O teste de Friedman^(21)^ foi utilizado para comparar as classificações dos escores entre os anos, enquanto que o teste de Mann-Kendall^(22-23)^ avaliou se houve tendência temporal estatisticamente significativa entre os anos avaliados. Adotou-se o nível de significância estatística de 0,05.

## RESULTADOS

Durante o período do estudo, foi registrado um total de 13.519 casos de câncer próstata recuperados dos RHC do estado do Espírito Santo, conforme pode ser observado na Figura 1.

**Figura 1:** Série histórica do número de casos de câncer de próstata diagnosticados de 2000 a 2020 registrados nos Registros Hospitalares de Câncer do estado do Espírito Santo (N=13.519)

A variável “sexo” foi a única variável sociodemográfica a apresentar 100% de completude, seguida da variável “idade”, que apresentou 0,26% de dados faltantes em 2016, e “procedência”, que teve incompletude variando de 0,20% a 2,34% entre os anos 2012 e 2019, portanto, classificadas como excelente em todo período estudado.

A variável “local de nascimento” obteve 5,91% de incompletude média no período, com destaque para os anos 2000, 2018 e 2020, apresentando, respectivamente, 14,29%, 12,45% e 16,88% de dados faltantes, sendo classificada como regular. Já a variável “raça/cor da pele” foi classificada como excelente ou boa na maioria dos anos estudados, porém, em 2006 e 2007, foi classificada como ruim, com 31,67% e 23,78% de incompletude, respectivamente. “Estado conjugal” foi uma variável com escore excelente ou bom em mais de 90% dos anos estudados, com destaque para os anos de 2012 e 2013, sendo classificada como ruim, apresentando incompletudes de 11,08% e 11,48%, respectivamente.

A variável” escolaridade” obteve escore excelente dos anos 2000 a 2004, com uma média de 2,74% de dados faltantes, porém, de 2005 a 2020, foi classificada na maioria dos anos como ruim, com destaque para o ano de 2010, sendo que quase 50% das observações eram ausentes. De modo semelhante, a variável “ocupação” apresentou média de 2,20% de incompletude de 2000 a 2004, e, a partir de 2005, foi classificada como ruim na maioria dos anos que seguem, obtendo, em 2018, 22,13% de dados faltantes e classificada como ruim. Ambas as variáveis “alcoolismo” e “tabagismo” apresentaram elevados índices de incompletude, sendo classificadas como muito ruim e ruim na maioria dos anos da série histórica de 2000-2020 estudada. Detalhes das classificações de completude ano a ano são apresentados na Tabela 1.

**Tabela 1:** Percentual de incompletude e classificação de completude das variáveis sociodemográficas dos Registros Hospitalares de Câncer referente aos casos de câncer de próstata da Rede de Atenção Oncológica do estado do Espírito Santo no período de 2000 a 2020 (N = 13.519)

Variável	2000	2001	2002	2003	2004	2005	2006	2007	2008	2009	2010	2011	2012	2013	2014	2015	2016	2017	2018	2019	2020
Sexo																					
Incompletude (%)	0,00	0,00	0,00	0,00	0,00	0,00	0,00	0,00	0,00	0,00	0,00	0,00	0,00	0,00	0,00	0,00	0,00	0,00	0,00	0,00	0,00
Escore	1	1	1	1	1	1	1	1	1	1	1	1	1	1	1	1	1	1	1	1	1
Idade																					
Incompletude (%)	0,00	0,00	0,00	0,00	0,00	0,00	0,00	0,00	0,00	0,00	0,00	0,00	0,00	0,00	0,00	0,00	0,26	0,00	0,00	0,00	0,00
Escore	1	1	1	1	1	1	1	1	1	1	1	1	1	1	1	1	1	1	1	1	1
Local de nascimento																					
Incompletude (%)	14,29	0,00	2,36	6,22	9,23	3,67	3,62	1,01	0,69	3,65	3,88	4,73	5,16	4,76	6,54	3,24	5,08	7,85	12,45	8,79	16,88
Escore	3	1	1	2	2	1	1	1	1	1	1	1	2	1	2	1	2	2	3	2	3
Raça/cor da pele																					
Incompletude (%)	1,59	2,38	0,47	0,00	0,38	10,73	31,67	23,78	9,17	7,81	5,00	12,05	11,29	14,50	6,92	4,32	2,01	1,57	2,57	3,46	3,43
Escore	1	1	1	1	1	3	4	4	2	2	2	3	3	3	2	1	1	1	1	1	1
Escolaridade																					
Incompletude (%)	3,17	2,38	2,83	2,22	3,08	29,66	30,77	29,68	32,87	45,02	49,12	26,54	23,55	26,91	26,07	25,09	19,79	12,13	18,87	7,41	12,16
Escore	1	1	1	1	1	4	4	4	4	4	4	4	4	4	4	4	3	3	3	2	3
Ocupação																					
Incompletude (%)	0,00	1,19	2,83	3,11	3,85	15,25	15,61	15,68	13,15	7,48	16,12	6,31	14,52	16,24	16,78	18,88	12,52	10,13	22,13	15,71	16,60
Escore	1	1	1	1	1	3	3	3	3	2	3	2	3	3	3	3	3	3	4	3	3
Procedência																					
Incompletude (%)	0,00	0,00	0,00	0,44	0,00	0,00	0,00	0,00	0,00	0,00	0,38	0,00	0,22	0,35	0,57	2,34	1,66	0,86	1,38	0,20	0,00
Escore	1	1	1	1	1	1	1	1	1	1	1	1	1	1	1	1	1	1	1	1	1
Estado conjugal																					
Incompletude (%)	0,00	2,38	0,00	0,89	0,00	7,63	3,85	4,05	5,88	6,98	6,12	9,47	11,08	11,48	3,03	3,60	1,31	3,42	1,88	2,08	2,29
Escore	1	1	1	1	1	2	1	1	2	2	2	2	3	3	1	1	1	1	1	1	1
Alcoolismo																					
Incompletude (%)	100,00	90,48	96,70	95,11	93,85	82,49	71,95	73,69	71,45	70,10	16,25	9,61	10,32	14,73	27,49	47,84	50,61	41,51	29,55	35,47	33,48
Escore	5	5	5	5	5	5	5	5	5	5	3	2	3	3	4	4	5	4	4	4	4
Tabagismo																					
Incompletude (%)	98,41	88,10	94,34	94,22	86,15	73,73	61,76	64,42	61,07	58,14	15,62	7,89	10,00	14,27	25,50	45,05	45,97	32,24	22,53	28,16	24,32
Escore	5	5	5	5	5	5	5	5	5	5	3	2	3	3	4	4	4	4	4	4	4

*1 - excelente; 2 - bom; 3 - regular; 4 - ruim; 5 - muito ruim.*

Em relação às variáveis clínicas, a variável “estadiamento TNM” foi classificada como muito ruim para todos os anos analisados, obtendo a pior completude em 2001, com 97,25% de dados ausentes. De forma semelhante, a variável “histórico familiar de câncer” também obteve escore muito ruim para todos os anos estudados, com destaque para o ano 2000, em que apresentou 100% de dados faltantes. Com classificação muito ruim e ruim para o período do estudo, a variável “estadiamento clínico do tumor por grupo (TNM)” apresentou incompletudes variando de 31,45% a 89,62%. Outra variável, o “estadiamento TNM patológico”, teve incompletude entre 14,88% e 39,53%, com escores variando entre regular e ruim.

A variável “estado da doença ao final do primeiro tratamento” no hospital, de 2000 a 2009, foi classificada como muito ruim, com uma média de 72,11% de observações faltantes, mas, a partir de 2010, apresentou melhores classificações, sendo ruim ou regular e uma média de 28,61% de incompletude. A variável “principal razão para a não realização do tratamento antineoplásico no hospital” variou seu escore de excelente a muito ruim no período estudado, com destaque para o ano de 2003, que apresentou incompletude de apenas 0,44%, e para o ano de 2006, atingindo quase 72% de ausência de dados. “Origem do encaminhamento” foi uma variável classificada como ruim e regular na maioria dos anos estudados, obtendo menores índices de incompletude ao final da série histórica, sendo que em 2020 apresentou 7,15% de dados ausentes. Já as variáveis “lateralidade do tumor primário” e “exames relevantes para o diagnóstico e planejamento da terapêutica do tumor” apresentaram escore excelente no início do período de estudo, passando a ser classificadas como ruim e muito ruim nos anos seguintes.

As variáveis “diagnóstico e tratamento anterior” e “data da triagem” apresentaram classificação excelente em quase todo período, mudando o escore para bom nos anos de 2006 e 2007 para a primeira variável em e 2012 e 2013 para a segunda. Já a variável “data do início do tratamento” foi classificada como excelente, exceto para os anos de 2009 a 2012 e 2018, em que seu escore foi bom ou regular.

As demais variáveis do banco apresentaram escore excelente em todos os anos estudados, com destaque para as variáveis “tipo de caso”, “data da primeira consulta”, “localização primária do tumor”, “localização primária detalhada”, “tipo histológico do tumor primário”, “Cadastro Nacional de Estabelecimentos de Saúde”, “Unidade Federativa da Unidade Hospitalar” e “município da Unidade Hospitalar”, que apresentaram 100% de completude. A Tabela 2 apresenta detalhadamente e cronologicamente a incompletude para as variáveis clínicas na série histórica estudada.

**Tabela 2:** Percentual de incompletude e classificação de completude das variáveis clínicas dos Registros Hospitalares de Câncer referente aos casos de câncer de próstata da Rede de Atenção Oncológica do estado do Espírito Santo no período de 2000 a 2020 (N = 13.519)

Variável	2000	2001	2002	2003	2004	2005	2006	2007	2008	2009	2010	2011	2012	2013	2014	2015	2016	2017	2018	2019	2020
Tipo de caso																					
Incompletude (%)	0,00	0,00	0,00	0,00	0,00	0,00	0,00	0,00	0,00	0,00	0,00	0,00	0,00	0,00	0,00	0,00	0,00	0,00	0,00	0,00	0,00
Escore	1	1	1	1	1	1	1	1	1	1	1	1	1	1	1	1	1	1	1	1	1
Data da primeira consulta																					
Incompletude (%)	0,00	0,00	0,00	0,00	0,00	0,00	0,00	0,00	0,00	0,00	0,00	0,00	0,00	0,00	0,00	0,00	0,00	0,00	0,00	0,00	0,00
Escore	1	1	1	1	1	1	1	1	1	1	1	1	1	1	1	1	1	1	1	1	1
Data do diagnóstico																					
Incompletude (%)	0,00	0,60	0,00	0,00	0,00	0,00	2,49	3,71	2,25	1,99	1,38	2,44	2,04	0,35	0,66	0,09	0,53	0,00	4,15	2,08	0,86
Escore	1	1	1	1	1	1	1	1	1	1	1	1	1	1	1	1	1	1	1	1	1
Diagnóstico e tratamento anterior																					
Incompletude (%)	4,76	0,00	0,00	0,00	0,00	0,00	7,92	5,23	0,35	1,00	2,12	1,00	1,18	0,81	0,38	0,27	0,61	1,00	3,66	1,09	0,14
Escore	1	1	1	1	1	1	2	2	1	1	1	1	1	1	1	1	1	1	1	1	1
Data do início do tratamento																					
Incompletude (%)	3,17	0,00	0,47	0,44	0,00	0,00	0,45	0,00	0,00	7,64	14,12	12,20	13,66	4,41	1,99	2,97	3,42	3,42	8,10	2,77	1,00
Escore	1	1	1	1	1	1	1	1	1	2	3	3	3	1	1	1	1	1	2	1	1
Data da triagem																					
Incompletude (%)	0,00	0,00	0,00	0,00	0,00	0,00	0,00	0,00	0,00	0,00	2,75	0,00	5,48	3,36	2,27	2,34	1,14	2,28	1,88	0,00	0,00
Escore	1	1	1	1	1	1	1	1	1	1	1	1	2	2	1	1	1	1	1	1	1
Base mais importante para o diagnóstico do tumor																					
Incompletude (%)	1,59	0,00	0,00	0,44	0,77	0,00	3,85	4,05	2,42	2,33	0,75	1,87	0,86	0,93	1,04	0,27	0,88	1,14	4,15	1,78	0,14
Escore	1	1	1	1	1	1	1	1	1	1	1	1	1	1	1	1	1	1	1	1	1
Localização primária do tumor																					
Incompletude (%)	0,00	0,00	0,00	0,00	0,00	0,00	0,00	0,00	0,00	0,00	0,00	0,00	0,00	0,00	0,00	0,00	0,00	0,00	0,00	0,00	0,00
Escore	1	1	1	1	1	1	1	1	1	1	1	1	1	1	1	1	1	1	1	1	1
Localização primária do tumor detalhada																					
Incompletude (%)	0,00	0,00	0,00	0,00	0,00	0,00	0,00	0,00	0,00	0,00	0,00	0,00	0,00	0,00	0,00	0,00	0,00	0,00	0,00	0,00	0,00
Escore	1	1	1	1	1	1	1	1	1	1	1	1	1	1	1	1	1	1	1	1	1
Tipo histológico do tumor primário																					
Incompletude (%)	0,00	0,00	0,00	0,00	0,00	0,00	0,00	0,00	0,00	0,00	0,00	0,00	0,00	0,00	0,00	0,00	0,00	0,00	0,00	0,00	0,00
Escore	1	1	1	1	1	1	1	1	1	1	1	1	1	1	1	1	1	1	1	1	1
Estadiamento TNM																					
Incompletude (%)	95,24	97,26	94,34	88,89	84,62	90,96	50,90	84,65	76,64	81,06	79,25	77,19	79,25	67,17	61,04	56,47	64,10	72,04	70,16	58,89	65,52
Escore	5	5	5	5	5	5	5	5	5	5	5	5	5	5	5	5	5	5	5	5	5
Estadiamento clínico do tumor por grupo (TNM)																					
Incompletude (%)	79,37	85,12	89,62	80,44	74,62	82,77	31,45	67,12	62,80	69,60	60,88	53,08	61,08	51,39	45,69	39,48	37,13	42,94	50,10	40,71	47,50
Escore	5	5	5	5	5	5	4	5	5	5	5	5	5	5	4	4	4	4	5	4	4
Outro estadiamento																					
Incompletude (%)	0,00	0,00	0,00	0,00	0,00	0,00	0,00	0,00	0,17	0,00	0,00	0,00	0,11	0,00	0,00	0,09	0,09	0,00	0,00	0,00	0,00
Escore	1	1	1	1	1	1	1	1	1	1	1	1	1	1	1	1	1	1	1	1	1
Estadiamento TNM Patológico																					
Incompletude (%)	19,05	21,43	25,47	21,33	30,00	33,05	18,55	16,69	21,97	39,53	26,75	38,59	36,34	31,32	29,10	33,54	34,41	16,41	36,36	15,42	14,88
Escore	3	4	4	4	4	4	3	3	4	4	4	4	4	4	4	4	4	3	4	3	3
Principal razão para a não realização do tratamento antineoplásico no hospital																					
Incompletude (%)	3,17	4,17	2,83	0,44	1,92	6,50	71,95	7,25	62,28	65,45	71,38	3,01	4,09	6,38	12,51	9,44	11,30	9,56	15,42	4,05	3,72
Escore	1	1	1	1	1	2	5	2	5	5	5	1	1	2	3	2	3	2	3	1	1
Primeiro tratamento recebido no hospital																					
Incompletude (%)	1,59	0,00	0,47	0,44	0,00	0,56	0,00	0,00	0,00	0,33	0,50	0,29	0,32	0,46	0,09	2,97	0,26	0,29	6,03	0,30	1,29
Escore	1	1	1	1	1	1	1	1	1	1	1	1	1	1	1	1	1	1	2	1	1
Estado da doença ao final do primeiro tratamento no hospital																					
Incompletude (%)	76,19	85,71	86,79	76,89	74,62	59,89	57,47	59,36	63,32	80,90	36,75	23,82	33,66	19,37	31,00	27,88	17,08	26,68	36,26	26,98	35,19
Escore	5	5	5	5	5	5	5	5	5	5	4	4	4	3	4	4	3	4	4	4	4
Data do óbito																					
Incompletude (%)	0,00	0,00	0,00	0,00	0,00	0,00	0,23	0,17	0,52	0,00	0,12	0,00	0,22	0,23	0,09	0,00	0,00	0,00	0,10	0,20	0,72
Escore	1	1	1	1	1	1	1	1	1	1	1	1	1	1	1	1	1	1	1	1	1
Histórico familiar de câncer																					
Incompletude (%)	100,00	97,02	99,53	96,44	97,69	87,85	83,48	85,50	73,70	71,43	79,88	69,58	74,84	64,50	74,60	72,75	77,58	73,04	71,84	68,38	58,23
Escore	5	5	5	5	5	5	5	5	5	5	5	5	5	5	5	5	5	5	5	5	5
Origem do encaminhamento																					
Incompletude (%)	30,16	37,50	24,06	13,33	11,92	13,28	26,24	18,55	21,63	17,77	21,88	13,77	19,03	14,62	16,02	7,82	12,08	17,69	8,89	8,50	7,15
Escore	4	4	4	3	3	3	4	3	4	3	4	3	3	3	3	2	3	3	2	2	2
Lateralidade do tumor primário																					
Incompletude (%)	0,00	0,00	0,00	0,00	0,38	0,00	0,45	6,07	12,11	4,65	6,38	10,62	9,46	11,02	12,61	8,99	6,57	2,57	10,08	1,38	4,29
Escore	1	1	1	1	1	1	1	2	3	1	2	3	2	3	3	2	2	2	3	3	2
Ocorrência de mais de um tumor primário																					
Incompletude (%)	0,00	0,00	0,00	0,00	0,00	0,00	0,00	0,00	0,00	0,00	2,75	0,00	5,48	3,36	2,27	2,25	1,05	2,28	1,88	0,00	0,00
Escore	1	1	1	1	1	1	1	1	1	1	1	1	2	1	1	1	1	1	1	1	1
Clínica do primeiro atendimento																					
Incompletude (%)	0,00	0,00	0,00	0,00	0,00	0,00	0,00	0,00	0,00	0,00	2,75	0,14	5,48	3,48	2,46	2,79	1,05	2,28	1,88	0,20	0,00
Escore	1	1	1	1	1	1	1	1	1	1	1	1	2	1	1	1	1	1	1	1	1
Clínica do início do tratamento																					
Incompletude (%)	0,00	0,00	0,00	0,00	0,00	0,00	0,00	0,00	0,00	0,00	0,00	0,00	0,32	0,46	0,19	0,36	0,35	0,43	5,93	0,20	0,86
Escore	1	1	1	1	1	1	1	1	1	1	1	1	1	1	1	1	1	1	2	1	1
Exames relevantes para o diagnóstico e planejamento da terapêutica do tumor																					
Incompletude (%)	0,00	0,60	0,00	0,00	4,23	1,13	19,00	18,04	24,39	25,42	12,50	6,17	8,06	5,10	9,76	10,70	4,12	3,85	9,49	7,91	10,59
Escore	1	1	1	1	1	1	3	3	4	4	3	2	2	2	2	3	1	1	2	2	3
Cadastro Nacional de Estabelecimentos de Saúde																					
Incompletude (%)	0,00	0,00	0,00	0,00	0,00	0,00	0,00	0,00	0,00	0,00	0,00	0,00	0,00	0,00	0,00	0,00	0,00	0,00	0,00	0,00	0,00
Escore	1	1	1	1	1	1	1	1	1	1	1	1	1	1	1	1	1	1	1	1	1
Unidade Federativa da Unidade Hospitalar																					
Incompletude (%)	0,00	0,00	0,00	0,00	0,00	0,00	0,00	0,00	0,00	0,00	0,00	0,00	0,00	0,00	0,00	0,00	0,00	0,00	0,00	0,00	0,00
Escore	1	1	1	1	1	1	1	1	1	1	1	1	1	1	1	1	1	1	1	1	1
Município da Unidade Hospitalar																					
Incompletude (%)	0,00	0,00	0,00	0,00	0,00	0,00	0,00	0,00	0,00	0,00	0,00	0,00	0,00	0,00	0,00	0,00	0,00	0,00	0,00	0,00	0,00
Escore	1	1	1	1	1	1	1	1	1	1	1	1	1	1	1	1	1	1	1	1	1

*1 - excelente; 2 - bom; 3 - regular; 4 - ruim; 5 - muito ruim.*

Com relação à comparação dos escores das variáveis epidemiológicas dos RHC do estado do Espírito Santo, o teste de Friedman mostrou que não houve diferença significativa (valor de *p* = 0,324) na classificação dos escores; portanto, a classificação foi semelhante entre 2000 e 2020.

Na Tabela 3, são evidenciadas pelo teste de Mann-Kendall as tendências significativas de decréscimo na incompletude para as variáveis “histórico familiar de câncer”, “alcoolismo”, “tabagismo”, “origem do encaminhamento”, “estadiamento TNM”, “estadiamento clínico do tumor por grupo (TNM)” e “estado da doença ao final do primeiro tratamento no hospital”. As variáveis “local de nascimento”, “clínica do primeiro atendimento”, “clínica do início do tratamento”, “procedência”, “lateralidade do tumor primário” e “ocupação” apresentaram tendência de acréscimo no índice de incompletude. As variáveis que apresentaram 100% de completude em todos os anos estudados não foram incluídas no teste de Mann-Kendall e, por isso, não aparecem na Tabela 3.

**Tabela 3:** Análise da tendência de incompletude das variáveis epidemiológicas e clínicas dos Registros Hospitalares de Câncer referente aos casos de câncer de próstata da Rede de Atenção Oncológica do estado do Espírito Santo no período de 2000 a 2020 (N = 13.519)

Variável	S	Valor de *p* ^*^	Tendência
Idade	12	0,364	Não significante
Local de nascimento	80	0,017	Acréscimo
Raça/cor da pele	-2	0,976	Não significante
Escolaridade	0	1,000	Não significante
Clínica do primeiro atendimento	73	0,018	Acréscimo
Clínica do início do tratamento	118	< 0,001	Acréscimo
Histórico familiar de câncer	-148	< 0,001	Decréscimo
Alcoolismo	-122	< 0,001	Decréscimo
Tabagismo	-128	< 0,001	Decréscimo
Procedência	81	0,008	Acréscimo
Origem do encaminhamento	-106	0,002	Decréscimo
Exames relevantes para o diagnóstico e planejamento da terapêutica do tumor	47	0,164	Não significante
Estado conjugal	29	0,397	Não significante
Diagnóstico e tratamento anterior	27	0,428	Não significante
Base mais importante para o diagnóstico do tumor	29	0,397	Não significante
Lateralidade do tumor primário	88	0,008	Acréscimo
Ocorrência de mais de um tumor primário	54	0,065	Não significante
Estadiamento TNM	-133	< 0,001	Decréscimo
Estadiamento clínico do tumor por grupo (TNM)	-134	< 0,001	Decréscimo
Outro estadiamento	17	0,477	Não significante
Estadiamento TNM patológico	14	0,695	Não significante
Principal razão para a não realização do tratamento antineoplásico no hospital	38	0,264	Não significante
Primeiro tratamento recebido no hospital	33	0,330	Não significante
Estado da doença ao final do primeiro tratamento no hospital	-126	< 0,001	Decréscimo
Ocupação	122	< 0,001	Acréscimo
Data do diagnóstico	37	0,271	Não significante
Data da triagem	54	0,065	Não significante
Data do início do tratamento	53	0,113	Não significante
Data do óbito	52	0,095	Não significante

* *Para significância, valor de p < 0,05.*

A Figura 2 mostra os gráficos da série histórica 2000 a 2020, com o percentual de incompletude das variáveis que apresentaram tendências significativas pelo teste de Mann-Kendall para o período estudado. As séries temporais com os dados de incompletude estão representadas pelas linhas contínuas, enquanto que as linhas tracejadas representam a tendência temporal.

**Figura 2:** Tendência de incompletude das variáveis sociodemográficas e clínicas, com tendência significativa pelo teste de Mann-Kendall dos Registros Hospitalares de Câncer referente aos casos de câncer de próstata da Rede de Atenção Oncológica do estado do Espírito Santo no período de 2000 a 2020 (N = 13.519)

## DISCUSSÃO

Os resultados mostraram que, no que tange aos casos de neoplasia prostática maligna do estado do Espírito Santo recuperados e analisados nos RHC, a maioria das variáveis epidemiológicas foi classificada como tendo uma completude excelente e/ou boa, destacando-se as variáveis “sexo”, “idade”, “procedência”, “data da primeira consulta”, “data do diagnóstico”, “diagnóstico e tratamento anterior”, “base mais importante para o diagnóstico do tumor”, “localização primária do tumor”, “localização primária detalhada do tumor”, “tipo histológico do tumor primário” e “primeiro tratamento recebido no hospital”. Entretanto, outras variáveis foram classificadas em alguns anos como regular e ruim, como “local de nascimento”, “raça/cor da pele”, “escolaridade”, “ocupação”, “estado conjugal”, “data do início do tratamento”, “exames relevantes para o diagnóstico e planejamento da terapêutica do tumor”. Além disso, houve fragilidade na informação de importantes variáveis clínico-epidemiológicas, com incompletudes acima de 50%, como “estadiamento TNM”, “estadiamento clínico do tumor por grupo (TNM)”, “história familiar”, além de “alcoolismo” e “tabagismo”. Corroborando os nossos resultados, estudo realizado com dados dos RHC do estado do Mato Grosso evidenciou que as variáveis “escolaridade”, “estadiamento TNM”, “histórico familiar de câncer”, “alcoolismo” e “tabagismo” exibiram incompletudes acima de 50%^(24)^.

As variáveis “sexo” e “idade” apresentaram completude classificada como excelente no banco analisado, como encontrado em outros estudos a partir de RHC de outros estados brasileiros^(13,15-16,24)^. Acredita-se que a baixa subjetividade interpretativa necessária para registrar essas informações corrobore a razão desse bom resultado.

A variável “local de nascimento” obteve 5,91% de incompletude, deixando-a com escore bom, porém apresentou uma tendência de acréscimo na incompletude no período analisado. Em outros estudos conduzidos em RHC do estado do Espírito Santo, essa variável apresentou incompletude média de 10,33%^(15)^ e 3,51%^(16)^.

“Raça/cor da pele” é uma variável importante no estudo do câncer de próstata, pois algumas etnias são fatores de risco para desenvolvimento desse câncer, como africana e asiática, apresentando maiores taxas de incidência e menor tempo de sobrevida para essa neoplasia^(5,25)^. Ou seja, essa variável transcende meramente uma distinção biológica. De fato, ela engloba uma complexidade que representa um conjunto de conotações econômicas e culturais, as quais denotam desigualdades no acesso à assistência médica, especialmente no âmbito do diagnóstico e tratamento do câncer. Nossas descobertas corroboram outras pesquisas realizadas no Brasil^(15,24,26-27)^. Importante destacar que a falta de completude na coleta dessa variável, aliada a possíveis registros equivocados, dificulta a obtenção clara da necessidade real de programas de promoção da saúde e prevenção de doenças em comunidades vulneráveis^(27)^. Adicionalmente, a variável que considera a raça/etnia ganha relevância ao ampliar os debates às iniquidades em saúde e vulnerabilidade individual, social e político-programática^(15,28-29)^.

“Escolaridade” foi classificada como ruim em mais de 50% do período de estudo, com incompletude média de 31%, um resultado semelhante ao encontrado por outros estudos^(12,15,24,27,30)^. O resultado encontrado nos RHC do Hospital Santa Rita de Cássia, único CACON do estado do Espírito Santo, apresentou 9,12% de dados faltantes, o que implica que os demais RHC do Espírito Santo possuem maiores incompletudes para essa variável^(16)^. Essa variável tem grande impacto no prognóstico do paciente, e sua baixa completude é de relevância clínica e epidemiológica^(15)^.

A variável “ocupação” apresentou, no início da série histórica, classificação excelente, porém, de 2005 a 2020, teve um aumento no percentual de dados ausente, passando à classificação regular, com 14,57% de incompletude média no período. Em estudo realizado em RHC de 21 estados brasileiros com relação à variável ocupação, foram identificados 46% de observações faltantes^(31)^. Outros estudo encontram percentuais semelhantes^(12,15-16,24,27)^.

As variáveis “estadiamento TNM”, “estadiamento clínico do tumor por grupo (TNM)” e “estadiamento TNM patológico” apresentaram escore de completude ruim ou muito ruim em quase todos os anos. Esses resultados corroboram outros estudos usando dados dos RHC pelo Brasil^(12,14-16,32)^. Por outro lado, estudo com banco de dados de um hospital público de São Paulo mostrou a variável “estadiamento TNM” com níveis excelentes de completude^(27)^. As variáveis de estadiamento são de extrema importância, pois fornecem informações da extensão da doença. Essas informações ajudam na definição do plano terapêutico à pessoa com câncer, o que facilita a padronização de procedimentos e a troca de experiências entre as instituições que oferecem tratamento oncológico^(11,15,24,33)^.

As variáveis “alcoolismo” e “tabagismo” foram classificadas como ruim ou muito ruim em quase todo o período do estudo. Esse é um resultado ruim, visto o potencial carcinogênico do álcool e tabaco^(34)^. Além disso, a variável “histórico familiar de câncer” também foi classificada como muito ruim em todos os anos do período, representando a marca de quase 80% de incompletude média. Provavelmente, isso ocorreu pois tratam-se de variáveis opcionais na ficha do tumor, e seu preenchimento varia substancialmente entre as instituições hospitalares. Tamanha incompletude é um fator preocupante, pois essa variável é um fato de risco para câncer de próstata^(2,5-6,35-37)^.

### Limitações do estudo

O presente estudo apresenta algumas limitações, como a utilização exclusivamente de dados obtidos de todos os RHC de um único estado brasileiro. Consequentemente, deve-se ter cautela ao interpretar os achados em relação à sua validade externa e generalização para os demais estados e regiões brasileiras. Embora os RHC forneçam informações valiosas sobre a qualidade dos serviços oferecidos, eles não representam de forma abrangente a epidemiologia do câncer regional ou nacional subjacente.

### Contribuições para as áreas da enfermagem, saúde ou políticas públicas

Para o melhor do nosso conhecimento, este é o primeiro estudo de uma série histórica recente que reporta completude das variáveis epidemiológicas dos RHC sobre os casos de neoplasia prostática malignas de toda a Rede de Atenção Oncológica Espírito Santo (ES) entre os anos de 2000 e 2020, trazendo informações valiosas para a Vigilância Epidemiológica e, especificamente, para a Vigilância do Câncer no território capixaba. Salienta-se que, em 80% dos países, existe uma tendência crescente da mortalidade prematura por câncer que está impactando o alcance da meta 3.4 dos Objetivos de Desenvolvimento Sustentável, que se refere à redução de, pelo menos, um terço da mortalidade prematura por doenças crônicas não transmissíveis até 2030^(38)^. Assim, evidencia-se a importância da implantação, manutenção, atualização e disponibilidade dos dados dos RHC para o melhor conhecimento do panorama do câncer para seu monitoramento e controle.

## CONCLUSÕES

Em síntese, verificamos que, de fato, a maioria das variáveis epidemiológicas revisadas dos RHC do estado do Espírito Santo, Brasil, foi classificada com completude excelente, embora importantes variáveis, como “estadiamento TNM” e “estadiamento clínico do tumor por grupo (TNM)”, tiveram elevados índices de incompletude para todos os anos entre 2000 e 2020. É premente a necessidade de dados consistentes e de alta qualidade dos RHC para melhor monitoramento das variáveis epidemiológicos da ficha do tumor. As contribuições dos RHC podem contribuir sobremaneira para a estruturação, formulação e planejamento de políticas públicas voltadas para a melhora do diagnóstico precoce, tratamento e qualidade de vida da população.
